# Are Nursing Students Trained to Meet the Needs of Cancer Survivors and Their Families? New Challenges, New Opportunities

**DOI:** 10.17533/udea.iee.v37n2e01

**Published:** 2019-09-19

**Authors:** Cristina García-Vivar, Virginia La Rosa-Salas, Marta Domingo-Oslé

**Affiliations:** 1 Nurse, Ph.D. Full University Professor and Vice-Dean of Research. Universidad de Navarra, Faculty of Nursing, Spain. Email: cgarvivar@unav.es Universidad de Navarra Universidad de Navarra Spain cgarvivar@unav.es; 2 Nurse, Ph.D. Assistant Ph.D. Professor and Director of the Practical Teaching Unit. Universidad de Navarra, Faculty of Nursing, Spain. Email: vlarsal@unav.es Universidad de Navarra Universidad de Navarra Spain vlarsal@unav.es; 3 Marta Domingo-Oslé. Nurse, Universidad de Navarra Clinic and Professor of the Practical Teaching Unit. Universidad de Navarra, Faculty of Nursing, Spain. Email: mdosle@unav.es Universidad de Navarra Universidad de Navarra Spain mdosle@unav.es

Current cancer treatments, along with more effective prevention measures, are producing increased cancer survival globally;(1) becoming - in many cases - a chronic disease.(2) Care of patients and families, living with a chronic disease, like cancer, constitutes one of the principal challenges for most health systems because they represent a heavy burden in terms of morbidity and mortality and carry a high percentage of the public expenditure in health.(3) Above all, the impact of cancer entails suffering and represents an important limitation in the quality of life, productivity, and functional state of the sick individuals and those living with them, that is, their family. More so, with evidence of the progressive increase of the number of older people with cancer, who are more prone to having comorbidities and other problems associated with their age, like dementia, depression, cerebrovascular accident, and diabetes.(4) Hence, the need for care and support toward these families is clear and imperative.

This new health context is influencing upon the setting where health care takes place. Thus, there is a need for Nursing and nurses to develop new ways of working that include innovative roles and profiles, greater openness of care approaches,(5) as well as the opportunity to demonstrate greater leadership in health services and, thereby, contribute to responding to current health challenges; among them, the challenge of chronicity and family-centered care.(6) In parallel manner, the new professional context is influencing on the formation of future health professionals, also in the setting of cancer care, as indicated by different national and European scientific societies on oncology.(7,8) Within these changing health, professional, and academic contexts, this editorial seeks to become a space for reflection, which highlights the training needs and acquisition of skills for nursing students, converted into health professionals, to care for cancer patients and their family members, not only during acute moments of the disease but throughout the different stages of cancer and with a comprehensive approach centered on the person and family.

Graduating students face a living, changing society with projection, which is why abilities and skills should be developed that permit them to synchronize their own theoretical knowledge as support of their clinical practice. However, how are these future nursing professionals being prepared to care for cancer survivors and their families? Where are the unique view and contribution by the nursing discipline to respond to the specific needs of these individuals? In Europe, the academic world, framed within the European Higher Education Area, faces the change of paradigm from a traditional approach of teacher-centered teaching to student-centered teaching. According to Miguel Díaz,(9) only thus is responsibility assumed on the training and development of their academic work. Thus, graduating students, future health professionals, will develop the necessary skills to “learn to learn” and, thereby, have the capacity to develop the skills they need for their dynamic work and guarantee continued education.

Given the complexity of oncology care, not only physical care, but psychosocial care inherent to the experience of living with cancer, it is vitally important to enhance nursing education in oncology. Enhancement based on introducing into the Nursing curriculum a set of innovative methodological strategies coherent with the results of learning sought, so that future nursing professionals obtain the knowledge and necessary skills to respond to the needs faced by people living with cancer. Namely, physical needs, such as management of pain, fatigue, or nausea and psychosocial needs, like management of fear and uncertainty, anxiety and depression, work and economic impact, interpersonal relationships and family communication and functioning, among others.(10) For significant learning by students that provide them the necessary tools to address the cited health needs, a variety of educational methodologies must be used, such as case studies, problem-based learning, project-based learning, cooperative learning, or the expository method or magisterial lesson. Also needed are more innovative evaluation methodologies, like the approach and resolution of clinical cases, simulation, or structured objective clinical evaluation. Likewise, participation is necessary from graduating students in the clinical practice in day centers, hospitals, and primary health care for long-term follow-up of cancer survivors. 

Lastly, identification of educational methodologies for significant student learning is closely related with nursing research on education. In this sense, we indicate the importance of educational decisions being based on the best evidence available to guarantee that future alumni have acquired the skills to care for patients and families living with cancer. The strength of nursing research lies in considering it an important tool to improve the clinical practice, but also as an asset to construct new frameworks of educational knowledge in benefit of student training to meet the health needs of society. 

We have new challenges and many opportunities in nursing research and education! 

## References

[1] Siegel RL, Miller KD, Jemal A (2017). Cancer Statistics, 2017. CA Cancer J. Clin.

[2] Granek L, Mizrakli Y, Ariad S, Jotkowitz A, Geffen DB (2017). Impact of a 3-Day Introductory Oncology Course on First-Year International Medical Students. J. Cancer Educ.

[3] Nekhlyudov L, Ganz PA, Arora NK, Rowland JH (2017). Going Beyond Being Lost in Transition: A Decade of Progress in Cancer Survivorship. J. Clin. Oncol.

[4] Bridges J, Wengström Y, Bailey DE (2016). Educational Preparation of Nurses Caring for Older People with Cancer: An International Perspective. Semin. Oncol .Nurs.

[5] Sánchez-Martín CI (2014). Cronicidad y complejidad: Nuevos roles en Enfermería. Enfermeras de Práctica Avanzada y paciente crónico. Enferm. Clin.

[6] García-Vivar C (2019). Family-centered care: a necessary commitment to address chronicity. Metas Enferm.

[7] Sociedad Española de Oncología Médica (SEOM). (2018). Informe SEOM: Formación de grado en Oncología: situación, retos y recomendaciones de futuro..

[8] European Oncology Nursing Society (EONS). (2018). Cancer Nursing Education Framework Contents.

[9] Díaz MDM (2006). Metodologías para optimizar el Aprendizaje. Segundo objetivo del Espacio Europeo de Educación Superior. Rev. Interuniversitaria de Formación Profesoral.

[10] Watson E, Shinkins B, Frith E, Neal D, Hamdy F, Walter F (2016). Symptoms, unmet needs, psychological well-being and health status in survivors of prostate cancer: implications for redesigning follow-up. BJU Int.

